# Utility of Next-Generation Sequencing in the Reconstruction of Clonal Architecture in a Patient with an *EGFR* Mutated Advanced Non-Small Cell Lung Cancer: A Case Report

**DOI:** 10.3390/diagnostics12051266

**Published:** 2022-05-19

**Authors:** Javier Simarro, Gema Pérez-Simó, Nuria Mancheño, Carlos Francisco Muñoz-Núñez, Enrique Cases, Óscar Juan, Sarai Palanca

**Affiliations:** 1 Molecular Biology Unit, Service of Clinical Analysis, Hospital Universitario y Politécnico La Fe, 46026 Valencia, Spain; javier_simarro@iislafe.es (J.S.); gema_perez@iislafe.es (G.P.-S.); 2 Clinical and Translational Cancer Research Group, Instituto de Investigación Sanitaria La Fe (IIS La Fe), 46026 Valencia, Spain; 3 Pathology Department, Hospital Universitario y Politécnico La Fe, 46026 Valencia, Spain; manchenyo_nur@gva.es; 4 Radiology Department, Hospital Universitario y Politécnico La Fe, 46026 Valencia, Spain; carlos.munoznunez@gmail.com; 5 Pulmonology Department, Hospital Universitario y Politécnico La Fe, 46026 Valencia, Spain; cases_enr@gva.es; 6 Medical Oncology Department, Hospital Universitario y Politécnico La Fe, 46026 Valencia, Spain; juan_osc@gva.es; 7 Biochemistry and Molecular Biology Department, Universidad de Valencia, Burjassot, 46100 Valencia, Spain

**Keywords:** non-small cell lung cancer, precision medicine, *EGFR* mutations, resistance mechanisms, molecular diagnostics, next-generation sequencing, liquid biopsy

## Abstract

EGFR tyrosine kinase inhibitors (EGFR-TKIs) have revolutionized the treatment of non-small cell lung cancer (NSCLC) patients with activating *EGFR* mutations. However, targeted therapies impose a strong selective pressure against the coexisting tumor populations that lead to the emergence of resistant clones. Molecular characterization of the disease is essential for the clinical management of the patient, both at diagnosis and after progression. Next-generation sequencing (NGS) has been established as a technique capable of providing clinically useful molecular profiling of the disease in tissue samples and in non-invasive liquid biopsy samples (LB). Here, we describe a case report of a patient with metastatic NSCLC harboring *EGFR* mutation who developed two independent resistance mechanisms (*EGFR*-T790M and *TP53* + *RB1* mutations) to dacomitinib. Osimertinib given as a second-line treatment eliminated the *EGFR*-T790M population and simultaneously consolidated the proliferation of the *TP53* + *RB1* clone that eventually led to the histologic transformation to small-cell lung cancer (SCLC). Comprehensive NGS profiling revealed the presence of the *TP53* + *RB1* clone in the pretreatment biopsy, while *EGFR*-T790M was only detected after progression on dacomitinib. Implementation of NGS studies in routine molecular diagnosis of tissue and LB samples provides a more comprehensive view of the clonal architecture of the disease in order to guide therapeutic decision-making.

## 1. Introduction

The development of targeted drugs against specific molecular aberrations has prompted the expansion of precision medicine in oncology [1]. Non-small cell lung cancer (NSCLC) patients harboring activating epidermal growth factor receptor (*EGFR*) mutations benefit from tyrosine kinase inhibitors (TKIs) with remarkable responses [2]. Despite the initial high response rate, resistance mechanisms will emerge as a consequence of the selective pressure of EGFR-TKI therapy against the multiple coexisting tumor populations [3].

The p.(Thr790Met) mutation in exon 20 of the *EGFR* gene is the major resistance mechanism to first- and second-generation EGFR-TKIs, being detected in 50–60% of patients with progressive disease [4]. Its detection has become a major challenge for the molecular diagnostics laboratories since the approval of Osimertinib, a third-generation EGFR-TKI, which has overcome this resistance mechanism by being highly effective in patients harboring p.(Thr790Met) [5]. However, various *EGFR* point mutations, tyrosine kinase receptors amplification, cell-cycle gene alterations, and lineage plasticity have been associated with Osimertinib resistance [6,7,8].

As a consequence of several treatment lines, the proliferation of certain tumor clones with individual molecular characteristics promotes a clonal architecture of the disease with clinical implications in terms of the emergence of resistant clones [9]. Based on the multiple resistance mechanisms to targeted therapies, comprehensive and dynamic molecular characterization of the disease is essential to provide the most updated information for patients’ clinical management [10].

In this sense, despite their limited sensitivity, liquid biopsies are a non-invasive approach to dealing with tumor heterogeneity and clonal architecture [11,12]. Circulating tumor nucleic acids released from both the primary tumor and metastatic sites to the systemic circulation represent the main cancer-derived material, and consequently, serially obtained samples may provide real-time information on the clonal composition of the disease [13,14].

To efficiently characterize tumors at the molecular level, single-gene approaches are being replaced by next-generation sequencing (NGS) because of its ability to simultaneously assess for diverse molecular alterations (point mutations, insertions, deletions, copy number variations and translocations) in a group of relevant genes [15,16]. This technique is able to board the tumor heterogeneity by quantifying tumor clones that harbor non-routinely assessed molecular alterations that could be responsible for future progression. Its application to both formalin-fixed paraffin-embedded (FFPE) samples and circulating tumor nucleic acids (ctNA) in liquid biopsies studies converts it into an essential tool for molecular diagnostics [17,18].

We herein report the utility of NGS studies in deciphering the clonal architecture of the disease in a patient with NSCLC exhibiting p.(Thr790Met) at progression to a second-generation EGFR-TKI, followed by a small-cell lung cancer (SCLC) transformation as a resistance mechanism to Osimertinib administered as a second-line treatment.

## 2. Case Report

A 68-year-old woman who quit smoking 15 years ago (30 packs/year) was diagnosed with stage-IV NSCLC adenocarcinoma (T3N3M1) in November 2014. Positron emission tomography–computed tomography (PET-TC) identified a 3 cm pulmonary mass in the upper left lobe. Adenopathies were detected in the aortopulmonary window and subcarinal and paratracheal lymph nodes. Bone metastases were identified in the left iliac crest, left femoral head and left scapula.

A biopsy specimen with 25% of tumor content was obtained through CT-guided bronchoscopy. A deletion in exon 19 of the *EGFR* gene was detected through Real Time PCR (Cobas^®^ EGFR Mutation Test v2 CE-IVD. Roche Diagnostics, Basel, Switzerland). Sanger Sequencing of exon 19 was conducted to confirm and characterize the mutation (*EGFR*-LRG_304t1: c.2235_2249del; p.(Glu746_Ala750del)).

In December 2014, the patient started first-line dacomitinib treatment (45 mg/day), a second-generation irreversible epidermal growth factor receptor tyrosine kinase inhibitor (EGFR-TKI), within a clinical trial (NCT01774721). CT evaluation scan in February 2015 indicated a partial response which was maintained as a stable disease until new pulmonary nodes were detected in a CT scan in October 2016 (Progression Free Survival (PFS): 22.2 months).

Due to disease progression, a liquid biopsy study was conducted through real-time PCR (Cobas^®^ EGFR Mutation Test v2 CE-IVD. Roche Diagnostics, Basel, Switzerland). This molecular study identified a deletion in exon 19 in concomitancy with the p.(Thr790Met) point mutation in exon 20, a well-known resistance mechanism to first and second-generation EGFR-TKI and a predictive biomarker of third-generation EGFR-TKI treatment benefit.

Consequently, Osimertinib (80 mg/day) was administered as second-line treatment, achieving a partial response maintained until the emergence of new bone and hepatic metastases in September 2017 (Osimertinib-PFS: 9.6 months). In order to characterize the resistance mechanism to second-line treatment and to explore new therapeutic approaches, a NGS study with Oncomine Lung cfDNA Assay (ThermoFisher Scientific, Waltham, MA, USA) was performed in liquid biopsy.

This study revealed the founder deletion in exon 19 of the *EGFR* gene found: c.2235_2249del; p.(Glu746_Ala750del) in a variant allele frequency (VAF) of 58%. Moreover, this mutation was found in concomitancy with a *TP53* deletion LRG_321t1: c.529_546del; p.(Pro177_Cys182del) in a VAF of 68%, while no evidence of p.(Thr790Met) was found in this sample.

The detection of a high VAF *TP53* mutation suggested a biallelic inactivation of this gene, which in concomitancy with the biallelic loss of *RB1* has been described as a virtually universal molecular event in SCLC [19]. In this sense, histologic transformation became a potential resistance mechanism to Osimertinib.

Since sequencing of the *RB1* gene is not included in Oncomine Lung cfDNA Assay, a Guardant360^®^CDx study was requested. This study added a truncating *RB1* mutation in a VAF of 68% [LRG_517t1: c.2047del; p.(Leu683Phefs*13)] to the known molecular profile. Taking together these results, liquid biopsy studies provided solid evidence of the resistance mechanism to second-line Osimertinib treatment in this patient.

Histologic characterization of a biopsy obtained from a novel hepatic metastasis confirmed the histologic transformation to SCLC. Subsequently patient received a third-line Cisplatin + Etoposide treatment with an initial partial response. Progressive disease with the emergence of a new adrenal gland and bone metastases was detected in the fourth month of treatment. The patient died in March 2018.

To further characterize diagnosis and SCLC-transformed biopsies, high throughput molecular techniques were conducted. Firstly, both samples were analyzed to search the *EGFR*-T790M clone with digital droplet PCR (ddPCR) and a PNA-Clamp TaqMan Assay with a limit of detection (LOD) of 0.1336% and 0.0996%, respectively. Neither of the assays could detect this variant in either of the samples.

**Figure 1:**
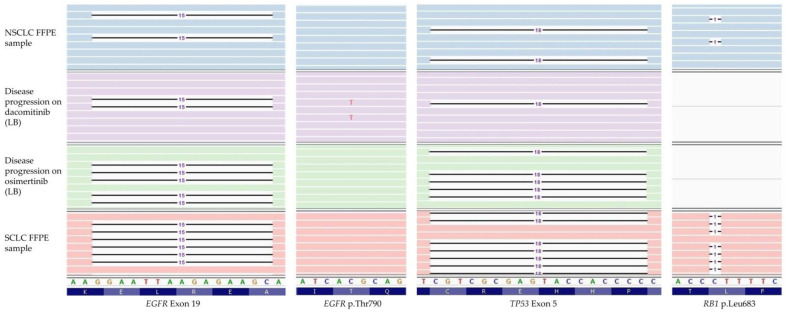
Integrative Genomics Viewer (IGV) browser visualization of the NGS results in the genomic positions, which became relevant for clinical management. For each sample and gene, colored bars represent the reads aligned along the reference genome (the *RB1* gene was only covered in the NGS studies of FFPE samples). Mismatched nucleotides are labeled. Black horizontal lines represent the nucleotides that have been deleted.

Moreover, to provide a broader molecular profile, both pretreatment and SCLC-transformed biopsies were retrospectively analyzed with Ampliseq Comprehensive Cancer Panel, a 409 gene NGS panel (ThermoFisher Scientific, Waltham, MA, USA). At progression, among other variants with unknown significance (VUS), SCLC transformed metastasis harbored *EGFR* (73%), *RB1* (96%) and *TP53* variants (90%). Interestingly, this NGS study revealed the presence of the *RB1* and *TP53* variants in the NSCLC pretreatment sample at low VAFs (3% and 2.5%, respectively) in concomitancy with the *EGFR* variant (20%) (Figure 1, Table 1).

**Figure 2:**
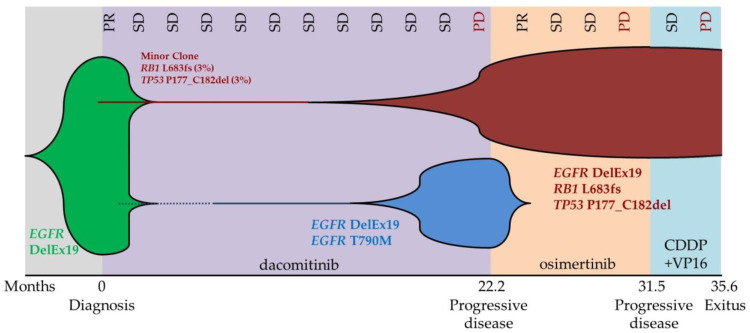
Clonal architecture of the disease was inferred through NGS analysis of FFPE and LB samples. The bottom horizontal axis represents the time from diagnosis and the subsequent treatment lines. The upper horizontal axis includes the results of the CT scans. PR: Partial Response; SD: Stable Disease; PD: Progressive Disease; CDDP + VP16: Cisplatin + Etoposide.

In order to recreate the clonal evolution of the disease, a liquid biopsy sample obtained after progression on dacomitinib was retrospectively analyzed with Oncomine Lung cfDNA Assay (ThermoFisher Scientific, Waltham, MA, USA). Apart from previously detected *EGFR* exon 19 deletion (VAF 7.9%) and *EGFR* p.(Thr790Met) mutations (VAF 3%), the *TP53* deletion was also detected in a VAF of 2.5% (Figure 2).

## 3. Discussion

In the last decade, NSCLC has become a paradigm of precision medicine in advanced cancer [20]. The development of drugs targeting specific molecular alterations has demonstrated a huge impact on patient clinical management [21]. Especially in *EGFR* mutated patients, tumor heterogeneity directly influences treatment response because of the evolution of resistance clones during tumor expansion [22].

Elucidating tumor heterogeneity may be crucial to infer clonal evolution to delay resistance or identify the best therapeutic approach after this event. For this purpose, molecular diagnostics laboratories must integrate cutting-edge approaches into their clinical routine.

In our patient, liquid biopsy samples provided clinically relevant information for treatment decisions when progressive disease to both EGFR-TKI treatment lines was revealed. The detection of *EGFR* p.(Thr790Met) after progression on dacomitinib provided a second line of treatment with a targeted drug. Subsequently, when the patient had disease progression on Osimertinib, a liquid biopsy study through NGS provided solid evidence of histological transformation as the resistance mechanism, which was subsequently confirmed in a new hepatic biopsy.

Moreover, in this case, retrospective NGS studies provided a more exhaustive and dynamic molecular profile, deciphering the tumor’s clonal architecture. In the pretreatment FFPE sample, NGS comprehensive panel revealed the presence of concomitant low-VAF *RB1* c.2047del; p.(Leu683Phefs*13) and *TP53* c.529_546del; p.(Pro177_Cys182del) mutations together with the dominant *EGFR* c.2235_2249del; p.(Glu746_Ala750del) mutation. The concomitancy of *RB1* and *TP53* in *EGFR* mutated patients has been described as a molecular signature that confers a higher risk of lineage transformation to SCLC [23,24]. This resistance mechanism has been described in approximately 10% of patients with disease progression after first- or second-generation EGFR-TKIs treatment, while its frequency as a resistance mechanism to Osimertinib as second-line treatment has been reported in up to 4–15% of patients.

Interestingly, the retrospective NGS study after progression on dacomitinib detected the *EGFR* p.(Thr790Met) and the *TP53* p.(Pro177_Cys182del) at similar VAFs. We hypothesized that the strong selective pressure enhanced the proliferation of two resistant tumor clones, which led to treatment failure; the *TP53* + *RB1* mutated clone, which was already present in the diagnostic FFPE sample, and the *EGFR* p.(Thr790Met) clone, which was not detected in the pretreatment FFPE sample by either NGS or ultrasensitive techniques (ddPCR and PNA Clamp TaqMan Assay).

The emergence of the *EGFR* p.(Thr790Met) clone could be explained by the acquisition model, which sustains that this variant is acquired as a response to the strong selective pressure imposed by first- and second-generation EGFR-TKI. However, we should not discard the selection model being this clone in an extremely low abundance or even being absent in tumor region which was biopsied [25].

After progression on Osimertinib, liquid biopsy studies detected the deletion in exon 19 of *EGFR* together with the *TP53* and *RB1* mutations. This molecular profile was also detected in the SCLC-transformed biopsy, which confirmed the biallelic loss of *RB1* (VAF: 96%) and *TP53* (VAF: 94%) and the permanence of the *EGFR* variant (VAF: 73%). Osimertinib should have eradicated the tumor clone harboring *EGFR* p.(Thr790Met) and, on the other hand, prompted the development of the clone with biallelic loss of *TP53* and *RB1,* which led to the histologic transformation to SCLC.

This clinical case reflects the remarkable heterogeneity of *EGFR* mutation-positive tumors, which may include minor clones harboring potential resistance mechanisms. Targeted treatment with EGFR-TKIs imposes a strong selective pressure on tumor cells prompting the clonal evolution of tumor populations. In this subgroup of high-risk patients, further research is needed to enhance the application of precision medicine. Although no targeted therapies have been approved for *TP53* and *RB1* mutations, combination treatment strategies at treatment onset have been reported in small cohorts or subgroup analyses of clinical trials. Combination therapies of EGFR-TKIs and chemotherapy, antiangiogenic agents or immunotherapy may be valuable options in the coming years in certain *EGFR* mutated patients [26,27,28].

High throughput molecular approaches in both FFPE and liquid biopsy samples allow a comprehensive, quantitative and dynamic understanding of genomic heterogeneity. Implementation of these techniques in the clinical routine may reflect the multiple adaptive changes in response to treatment and lead to a personalized molecular-guided treatment decision to delay or react against the radiological progression.

## Figures and Tables

**Table 1:** Variant allele frequencies of the *EGFR*, *TP53* and *RB1* variants detected in NGS studies.

Moment	*EGFR* Exon 19	*EGFR* p.(Thr790)	*TP53* Exon 5	*RB1* p.(Leu683)
NSCLC FFPE sample	p.(Glu746_Ala750del) 20%	NMD	p.(Pro177_Cys182del) 2.5%	p.(Leu683Phefs*13) 3%
Disease progression on dacomitinib (LB)	p.(Glu746_Ala750del) 7.9%	p.(Thr790Met) 3%	p.(Pro177_Cys182del) 2.5%	NI
Disease progression on osimertinib (LB)	p.(Glu746_Ala750del) 58%	NMD	p.(Pro177_Cys182del) 68%	NI
SCLC FFPE sample	p.(Glu746_Ala750del) 73%	NMD	p.(Pro177_Cys182del) 90%	p.(Leu683Phefs*13) 96%

NSCLC: Non-small cell lung cancer; FFPE: formalin-fixed paraffin-embedded; SCLC: Small cell lung cancer; NMD: No Mutation Detected; LB: Liquid Biopsy; NI: Not Included.
